# Diagnostic Accuracy of Lung Ultrasound in Rabbit Subclinical Lung Lesions

**DOI:** 10.3390/vetsci12040340

**Published:** 2025-04-07

**Authors:** Roberto Sargo, Inês Tomé, Filipe Silva, Mário Ginja

**Affiliations:** 1 Veterinary Teaching Hospital, University of Trás-os-Montes e Alto Douro, 5000-801 Vila Real, Portugal; inestome@utad.pt (I.T.); fsilva@utad.pt (F.S.); mginja@utad.pt (M.G.); 2 CECAV-Veterinary and Animal Science Research Centre and AL4AnimalS—Associate Laboratory for Animal and Veterinary Science, University of Trás-os-Montes e Alto Douro, Quinta de Prados, 5000-801 Vila Real, Portugal; 3 Department of Veterinary Science, University of Trás-os-Montes e Alto Douro, 5000-801 Vila Real, Portugal

**Keywords:** lung ultrasound (LUS), computed tomography, B-lines, non-invasive imaging

## Abstract

Rabbits are commonly affected by subclinical lung diseases. Computed tomography (CT) is the gold standard for diagnosing rabbit lung diseases but is not widely available and requires anesthesia, delaying diagnosis. Lung ultrasound (LUS) has emerged as a radiation-free, bedside diagnostic tool in human and veterinary medicine, though its use in rabbit medicine is not routine. This study aimed to evaluate LUS for detecting subclinical lung lesions in rabbits. Thirty healthy, five-month-old male New Zealand white rabbits underwent lung ultrasound, exploring four regions in each hemithorax, followed by thoracic CT under sedation with midazolam and butorphanol. The ultrasound images were scored as positive or negative, and the CT exams were assessed for aeration using threshold masks. The results showed that 63% of rabbits had one or more affected regions in the ultrasound images, and 19% of the regions were positive. CT identified 54% of the regions as positive for poorly aerated tissue, with 26/30 rabbits showing at least one positive region. The sensitivity and specificity of LUS were 33.33% and 93.48%, respectively, with an accuracy of 67.92% for detecting subclinical lesions. While LUS demonstrated a high specificity, its sensitivity was low compared to CT, highlighting the need for further refinement in its use for rabbit respiratory disease diagnosis.

## Simple Summary

Lung ultrasound is a cost-effective, radiation-free diagnostic tool that is easy to learn and can be used at the bedside. While it is commonly used in human and veterinary medicine, its application in small mammals like rabbits is not routine. This study aimed to evaluate the accuracy of lung ultrasound in diagnosing subclinical lung lesions compared to computed tomography (CT). Although the accuracy was around 68%, lung ultrasound showed promise as a tool for ruling out disease, especially when a negative result was found.

## 1. Introduction

Respiratory diseases are commonly responsible for morbidity and mortality in pet rabbits [1,2,3,4]. Anatomy and prey behavior play an important role in the masking of overt signs of disease, making diagnosis difficult in the early stage of diseases. Rabbits are obligatory nasal breathers and have a small thoracic cavity. At rest, their respiratory movements are very shallow and they seem to depend more on diaphragmatic movements, meaning that an increase in intra-abdominal pressure can cause marked dyspnea [3,4,5]. Their resting respiratory rate is between 30 and 60 respirations per minute and usually increases during veterinary practice due to the stress generated by travel and being in a non-familiar environment [6]. Rabbit lungs are not lobulated, meaning that pneumonia has a lobar distribution [1,3,4,5,6]. The right lung is divided into four lobes (cranial, medial, caudal, and accessory), while the left lung is only divided into two lobes (cranial and caudal) [4,6], but mediastinal fat is superimposed on small cranial lobes, limiting its expansion [4]. Subclinical respiratory disease is often present and goes unnoticed during clinical exams, which can complicate anesthetic procedures [1,7].

Thoracic radiography serves as the primary method for screening lower-respiratory-tract diseases; however, it has certain limitations, especially when it comes to accurately detecting and characterizing lung lesions [4,8].

Computed tomography (CT) is regarded as the gold standard for diagnosing lung disease in rabbits [4,9,10]. However, there is limited research available on normal tomographic images of rabbit lungs [9,11]. High-quality CT imaging requires the use of sedation or anesthesia [1,8,12], but a recent study indicates that anesthesia can significantly decrease lung volume and increase lung attenuation in rabbits, suggesting sedation as a preferable alternative [11]. Additionally, the mean lung attenuation in sedated rabbits is relatively high compared to that of dogs, which can make the interpretation of results more challenging [11,13]. Semi-quantitative CT imaging tools have been utilized to enhance the detection of ground-glass opacities (GGO) in human patients [14,15]. These methods rely on measuring different Hounsfield units (HUs) within the pulmonary parenchyma and generating color-coded, threshold-based masks to highlight areas with varying attenuation [14,16]. Using these approaches, early or mild lesions of COVID-19 could be diagnosed before symptoms arise, aiding in the prognosis assessment of patients [17].

Although CT is the most effective diagnostic tool for detecting lung diseases, it is expensive, is not widely accessible, and involves ionizing radiation exposure. These factors make CT less likely to be the first choice for clinicians when subclinical disease is suspected.

Lung ultrasound (LUS) has gained significant attention in recent years following the SARS-CoV-2 pandemic. However, its use in both human and veterinary settings has been established since the early 2000s [18,19].

Veterinary thoracic ultrasonography has advanced in recent years, becoming a frequently employed technique in emergency situations for the rapid assessment of pleural or pulmonary fluid in unstable animals [19,20,21,22]. It is also used in stable animals with suspected pulmonary disease [23,24]. Studies conducted in rats have demonstrated the potential of thoracic ultrasonography for detecting acute respiratory distress syndrome, while other studies have shown its ability to detect pulmonary edema in various animal species [25]. The use of thoracic ultrasonography in rabbits is primarily limited to the evaluation of the thymus, heart, and the presence of pleural effusions. Additionally, it is indicated to assist in the cytological assessment of intrathoracic structures [1,10,12]. More recently, Zhu et al. demonstrated the utility of LUS in the diagnosis of pulmonary edema [26].

Lung ultrasound is radiation-free, versatile, patient-adaptable, easy to learn to perform, and cost-effective [27]. These attributes make it an ideal tool for triaging patients with dyspnea.

The purpose of this study is to assess the accuracy of lung ultrasound in diagnosing subclinical lung diseases in rabbits and to evaluate its potential as a screening tool in clinical practice. To the authors’ knowledge, no previous studies have been specifically dedicated to this topic. Computed tomography, considered the gold standard, was chosen as the reference method for comparison to determine its diagnostic accuracy.

## 2. Materials and Methods

### 2.1. Study Design and Subject Inclusion

A prospective study was designed to supplement the use of animals from a separate study that focused on CT imaging of the hip joints. Thirty, healthy, male, five-month-old New Zealand white rabbits were used in this study. The number of animals included in the experiment was determined to achieve a statistical power of 0.8 (80%), ensuring a high probability of detecting a true effect while minimizing the risk of Type II errors (false negative) [28]. All procedures complied with both European and national regulations on the protection of animals used in scientific research, following the European Directive 2010/63/EU and National Decree-Law 113/2013. The study received approval from the relevant Portuguese authority, the General Directorate for Food and Veterinary (DGAV_0421/000/000/2022).

Animals were housed individually or in pairs in cages measuring 80 cm × 55 cm × 55 cm. All cages had an elevated platform and were cleaned every day. The animals were housed in a ventilated room with a controlled temperature of 19–21 °C and relative humidity maintained at 50–60%. A 12 h light/dark cycle was implemented using controlled artificial lighting. The animals were provided with a standard pellet diet and had unrestricted access to water. The animals were weighed every other day and underwent a clinical examination by a veterinarian.

### 2.2. LUS Protocol

Animals were positioned in the sphinx position and held on a table with minimal handling for ultrasound. Their fur was parted with alcohol, and a multifrequency linear array transducer (L8-18i, General Electric Medical Systems, Buc, France) on a Logiq P9 ultrasound machine (General Electric Medical Systems, Buc, France) was used with a musculoskeletal preset at 15 MHz with a depth of 3 cm. The focus was set at the level of the pleural line. All the exams were made in 2D (two-dimensional) mode, and 3 s video loops were saved for further evaluation.

Lung ultrasound was conducted by one operator (RS) with more than 10 years of experience in echocardiography and associated lung ultrasound. The protocol was adapted from the Vet BLUE protocol [29]. In rabbits, the lungs extend from the 3rd to the 11th intercostal space on the right side and from the 4th to the 10th intercostal space on the left side [9]. Each lung was examined using a probe held perpendicular to the rib cage, focusing on four distinct locations. The examination began in the caudodorsal region, just above the xiphoid process in the dorsal third of the thorax, starting at the diaphragmatic border and covering the adjacent lung across three intercostal spaces. The probe was then slid across three intercostal spaces cranially to assess the perihilar region, maintaining the same level as the caudodorsal region. Next, the probe was moved three intercostal spaces cranially and positioned at the level of the shoulder to examine the cranial lung lobe (cranial region). It was subsequently moved three intercostal spaces caudally to evaluate the middle lung region (middle region). This procedure was repeated on the contralateral lung.

### 2.3. Thoracic CT Protocol

Following the lung ultrasound examination, the rabbits were sedated with butorphanol (Butomidor^®^, Richter Pharma AG, Wels, Austria; 0.4 mg/kg, IM) and midazolam (Dormazolan^®^, Le Vet Beheer B.V., Oudewater, The Netherlands; 0.5 mg/kg, IM) and positioned in the prone position inside a cardboard box that was fixed to the CT bed. Their positioning was reassessed after ten minutes before proceeding with the CT scan.

CT scans were acquired in a cranial-to-caudal direction using a lung algorithm. The scanning protocol was set with a tube current of 80 mA and tube voltage of 100 kVp. Slice thickness was set at 1.25 mm, with a rotation time of 0.98 s and a pitch of 1.38. A 16-slice CT scanner (Revolution™ ACT, General Electric Medical Systems, Amersham, UK) was used for image acquisition.

### 2.4. Image Analysis

**Figure 1:**
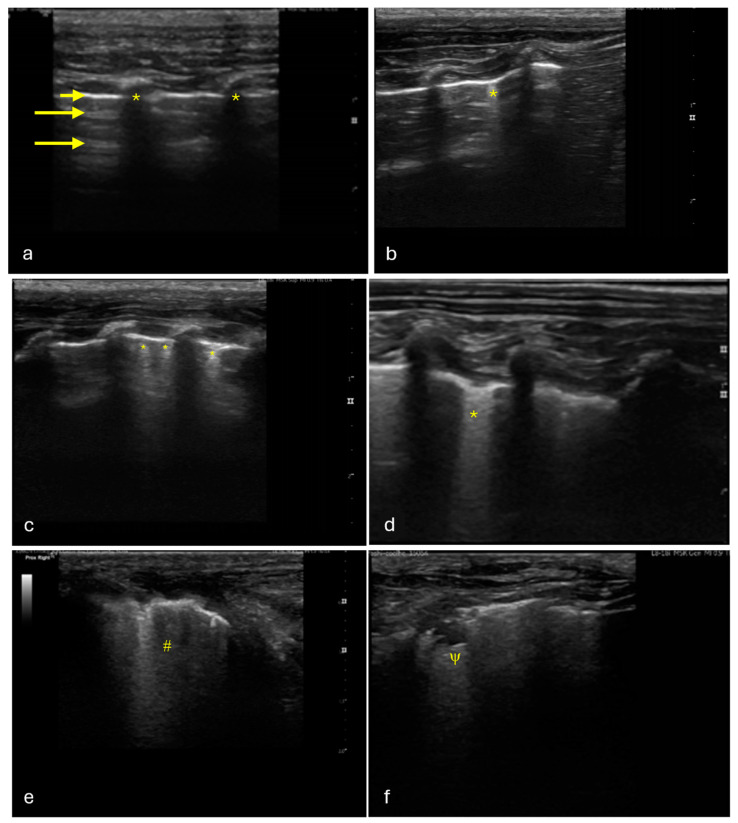
Lung ultrasound images of different regions of a study rabbit’s lung, showing identified patterns observed during the study: (**a**) Normal lung—air-filled zones showing the rib acoustic shadows (*), the hyperechoic pleural line (short arrow), and A-line artifacts representing horizontal reverberation artifacts of the hyperechoic pleural line (long arrows). (**b**) B-line (*) vertical, hyperechoic artifact that extends from the pleural line to the bottom of the screen without fading. (**c**) Multiple B-lines (*). (**d**) Coalescent B-lines (*). (**e**) Tissue sign (#), hepatization-like appearance of the lung. (**f**) Shred sign (ψ), subpleural consolidation with an irregular or “shredded” border between the aerated and consolidated lung. Ultrasound was performed using a musculoskeletal preset (15 MHz), with a depth of 3 cm and the focus set at the level of the pleural line.

Lung ultrasound images were assessed in real time during the examination and later reviewed for confirmation using the embedded software of the ultrasound machine. Data were scored as follows: 0 (no artifacts observed), 1 (a single B-line visible), 2 (two or three B-lines present), 3 (more than three B-lines or confluent B-lines detected), 4 (tissue sign observed), 5 (shredding sign noted), 6 (pleural effusion identified), or 7 (pneumothorax detected) (Figure 1). Scores of 0 and 1 were considered negative and all others were considered positive [19].

CT scans were reviewed using an open-source software, 3D Slicer, designed for the visualization, processing, segmentation, and analysis of images [30].

**Figure 2:**
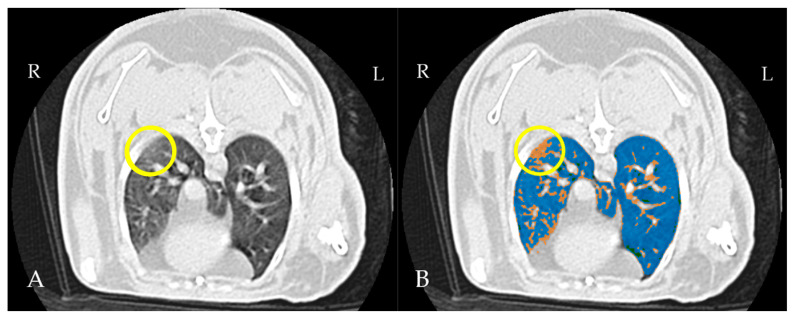
Thoracic CT scan in transverse plan of a rabbit. (**A**) Normal view, a circle marks an area of increased attenuation in the periphery of the right lung, caudodorsal region. (**B**) Threshold masking highlights an area of poorly aerated tissue. Aerated tissue is shown in blue (−899 to −500 HU), while infiltrated lung/ground-glass opacity (GGO) is shown in orange (−499 to −101 HU).

The raw data were uploaded into the 3D Slicer interface, and masks based on specific Hounsfield unit (HU) thresholds were applied to distinguish anatomical structures and outline their boundaries. The following Hounsfield unit (HU) thresholds were applied: 350 HU to −100 HU for the abdominal soft tissues, mediastinum, lung vasculature, and thoracic wall; −1050 HU to −900 HU for the trachea and primary trough tertiary bronchi; and −899 HU to −101 HU for the lung parenchyma [13,31]. The Lung CT Segmenter extension was employed to produce semi-automated segmentation of both lungs. The volume was then imported into Lung CT Analyzer, which utilized the obtained segmentation to categorize the lung parenchyma in different regions: emphysema (−1050 to −900 HU), normal aerated lung parenchyma (−899 to −500 HU), ground-glass opacity (GGO)/infiltrated lung parenchyma (−499 to −101 HU), and vessels and collapsed lung (−100 to 1000 HU) [13,32]. A cut-off was set at −500 HU for normal aerated lungs, following published data for dogs [13,30,33] (Figure 2).

Thoracic regions were divided into caudodorsal, perihilar, cranial, and medioventral sections, following the scheme used for the LUS protocol. The different regions were analyzed using mask filters. GGO and consolidations were easily identified. The ones that extended into the pleural line were assigned a value of 1 or 2, respectively, and were registered as positive. Regions that did not have visible GGO or consolidations or whose GGO or consolidations did not reach the pleural line were considered as negative and assigned a value of 0.

## 3. Statistical Analysis

The data acquired from both protocols were recorded using Microsoft Excel for Windows.

Descriptive statistics were applied to each variable, which included calculating the mean, standard deviation, and range.

To evaluate diagnostic performance, sensitivity, specificity, positive predictive value (PPV), and negative predictive value (NPV) were calculated using the following formulas.

Sensitivity = TP/(TP + FN),(1)

Specificity = TN/(TN + FP)(2)

PPV = TP/(TP + FP)(3)

NPV = TN/(TN + FN)(4)

The accuracy was calculated as the proportion of correctly classified cases (true positives and true negatives) among all cases as follows:

Accuracy = (TP + TN)/(TP + TN + FP + FN)(5)

The confidence intervals for all metrics were computed using JMP 11, with the level of statistical significance set at *p* ≤ 0.05.

We defined a true positive (TP) as being CT- and LUS-positive, a true negative (TN) as being negative on both imaging modalities, a false positive (FP) as LUS-positive and CT-negative, and a false negative as LUS-negative and CT-positive.

## 4. Results

### 4.1. Study Population

Thirty, male, 5-month-old New Zealand white rabbits were included in this study. All animals were successfully submitted to lung ultrasound and thoracic computed tomography protocols. In total, 240 lung regions were evaluated in each protocol.

### 4.2. Thoracic LUS

The LUS exams identified the presence of abnormal artifacts, including B-lines, consolidation, and shredding signs. Of the 240 regions assessed, 181 (75.4%) showed no visible abnormal artifacts; 14 (5.8%) had only a single B-line; seven (2.9%) had two or three B-lines; 29 (12.1%) had more than three B-lines or confluent B-lines; four (1.7%) had consolidation tissue signs; and five (2.1%) exhibited consolidation shredding signs (Table 1).

Of the 30 rabbits, 19 (63.3%) had one or more regions that were positive on the LUS. A Pearson chi-square test was conducted to assess the relationship between the hemithorax and LUS findings. The right side was significantly more affected, with 29 out of 120 regions (24.2%) showing positivity compared to 14 out of 120 regions (11.7%) on the left side: χ^2^(1, N = 240) = 6.375 at *p* = 0.0116.

The same statistical test was used to assess the relationship between the evaluated region and LUS findings. The region significantly affected the findings (χ^2^(7, N = 240) = 23.997; *p* = 0.0011), with the right medial region (13/30) and the right cranial region (8/30) being more affected than the others.

### 4.3. Thoracic CT

This imaging modality identified GGOs in 102 out of 240 lung regions and only three areas of consolidation. A total of 135 lung regions were considered normal (Table 2).

The lung CT evaluation showed that 86.6% (26/30) of the rabbits had areas of increased attenuation. A Pearson chi-square test was conducted to assess the relationship between the hemithorax side and the presence of increased attenuation. The right hemithorax was found to have significantly more areas of increased attenuation (59/120) compared to the left one (43/120): χ^2^(1, N = 240) = 4.37 at *p* = 0.037.

Regarding the defined regions, the right medial region (17/30) and the right cranial region (19/30) were found to have more areas of increased attenuation compared to the other regions. A Pearson chi-square test revealed a significant difference between the regions: χ^2^(7, N = 240) = 23.997 at *p* = 0.0011).

### 4.4. Performance of LUS

The sensitivity was calculated, and a value of 33.33% (95% CI: 24.31% to 43.36%) was obtained.

The specificity yielded a value of 93.48% (95% CI: 87.98% to 96.97%).

The positive predictive value (PPV) was calculated, and the result was 79.07% (95% CI: 65.48% to 88.27%).

The negative predictive value (NPV) was also calculated, and a value of 65.48% (95% CI: 62.16% to 68.66%) was obtained.

The accuracy was calculated as the proportion of correctly classified cases (true positives and true negatives) among all cases, and a value of 67.92% (95% CI: 61.61% to 73.78%) was obtained.

LUS identified 89% of the consolidations detected in the CT images, including 100% of the areas presenting a shredding sign (Table 3).

## 5. Discussion

Rabbit respiratory diseases are common in pet, laboratory, and farmed animals and are well documented in the literature [1,2,3,4,34,35]. Even in controlled environments, like laboratory facilities, respiratory infections by bacteria belonging to the genus Bordetella and Pasteurella are frequently found [35]. These diseases frequently remain undetected until they progress to an advanced stage. The high prevalence of subclinical cases underscores the importance of early diagnosis, as they also present a significant risk for complications related to anesthesia [1,7].

The aim of this study was to assess the accuracy of LUS in diagnosing subclinical lung disease in rabbits. LUS was easily performed in the animals that were awake, only requiring them to be manually restrained; however, it was less effective in detecting subclinical lung lesions compared to CT. With an accuracy of 67.92%, LUS correctly classified approximately two-thirds of the lung regions when compared to CT, which served as the gold standard for detecting subclinical lung lesions.

Although the stethoscope remains the primary tool for assessing lung diseases, conventional radiography is the main diagnostic tool for confirmation. However, it has significant limitations in accurately identifying and characterizing lesions [4,8]. CT is considered the gold standard in lung diagnostic imaging [4,6,8], but little research has been conducted in rabbits. Published studies on lung CT protocols are scarce and focused on healthy laboratory animals; nonetheless, lung lesions have been reported in one of these studies [9,11]. Ground-glass opacities are described as areas with a hazy increase in lung opacity but with the bronchial and vessel margins preserved. When the bronchial tree and vessels are obscured, it is considered consolidation [36]. GGOs are the reflection of different conditions that affect the lungs and result in the alveolar filling with fluid or debris or in thickened alveolar walls or interstitia [37]. Semiquantitative imaging techniques are gaining interest in both veterinary and human medicine as they help identify subtle changes in lung attenuation that are difficult to discern with the naked eye, making the diagnosis of early to mild disease before the onset of typical clinical signs faster and more accurate [13,14,15,16,17]. The defined cutoff of 500 HU is intended to separate aerated tissue from poorly aerated tissue, helping the software to accurately identify areas with increased attenuation [13].

In our study, 86.6% of the rabbits (26/30) had lung areas with high attenuation values in the CT exams, consistent with ground-glass opacities or consolidation. These were more prevalent in the right cranial and medial areas. Although the animals were sedated and not anesthetized, they remained in the prone position for 10 min prior to the CT exam, which could have led to an increase in attenuation, as it is seen in dogs, in the ventral portions of the cranial and medial lung lobes [13,32]. Nevertheless, these same areas were also more prone to lesion detection when using the LUS protocol, albeit to a lesser extent. The presence of B-lines was also more consistently found in the middle lobes of dogs and cats with alveolar interstitial syndrome in a study [19]. Consistently, the right lung was more prone to the presence of lung lesions both on CT and LUS. A previous study demonstrated that the right lung exhibited a higher mean attenuation value than the left lung on CT exams of sedated rabbits [11]. With the exception of aspiration pneumonia, which more commonly affects the right lung, other lung diseases do not typically show a marked predisposition for either lung [38,39].

While the high specificity (93.48%) suggests that LUS is highly effective at ruling out regions without lesions, the relatively low sensitivity (33.33%) indicates limitations in detecting regions with subclinical lesions. This discrepancy suggests that LUS may under detect certain subtle lung abnormalities, potentially due to its lower sensitivity to mild or early-stage lesions compared to CT. These results reflect the ones found for dogs, in which the application of the VETBLUE protocol had a high specificity but a low sensibility for alveolar-interstitial-syndrome-related abnormalities [19]. As found in the previous study, in our study, LUS identified the consolidated areas more accurately, showing a better performance in identifying these types of lesions.

This study has several limitations, with the number of observers being the most significant. Future studies should include trained clinicians with expertise in both LUS and CT interpretation. While CT remains the gold standard for diagnosing lung diseases, it requires sedation or anesthesia for optimal positioning [1,8,12]. Although previous studies have shown minimal to no effect of sedation on lung attenuation in dogs [13,32], the rabbits in this study were not fasted, and the potential impact of increased abdominal pressure from a filled stomach on lung volume and the attenuation of dependent lung regions should be considered.

## 6. Conclusions

This study demonstrates that lung ultrasound (LUS) can detect subclinical lung abnormalities in rabbits, identifying pathological artifacts such as B-lines, consolidation, and shredding signs. Our results suggest that LUS is highly specific for identifying lung pathology; however, its moderate sensitivity indicates that some lung abnormalities may be missed, highlighting the need for complementary imaging when necessary.

## Figures and Tables

**Table 1:** LUS scoring by scanned region. A total of 240 lung regions were assessed using lung ultrasound (LUS), with scores assigned based on aeration patterns.

LUS Scoring	Number of Regions	Percentage %
0	181	75
1	14	6
2	7	3
3	29	12
4	4	2
5	5	2

**Table 2:** CT scoring by scanned region. A total of 240 lung regions were assessed using computed tomography (CT), with scores assigned based on aeration patterns and the presence of lung abnormalities.

CT Scoring	Number of Regions	Percentage %
0	135	56.25
1	102	42.5
2	3	1.25

**Table 3:** Comparison of LUS and CT findings by lung region. The table shows the distribution of positive and negative CT findings across LUS scores (0–5) for a total of 240 lung regions.

				LUS			
		0	1	2	3	4	5
CT	Positive	63	5	6	9	3	5
	Negative	118	9	1	20	1	0

## Data Availability

Data are available on request from the authors.

## Abbreviations

The following abbreviations are used in this manuscript:

CT	Computed tomography
FN	False negatives
FP	False positives
GGO	Ground-glass opacities
HU	Hounsfield unit
LUS	Lung ultrasound
NPV	Negative predictive value
PPV	Positive predictive value
TN	True negatives
TP	True positives

## References

[1] Hedley J., Meredith L. (2014). Respiratory Disease. BSAVA Manual of Rabbit Medicine.

[2] Lennox A.M., Mancinelli E., Quesenberry K.E., Orcutt C.J., Mans C., Carpenter J.W. (2020). 15—Respiratory Disease. Ferrets, Rabbits, and Rodents.

[3] Johnson-Delaney C.A., Orosz S.E. (2011). Rabbit Respiratory System: Clinical Anatomy, Physiology and Disease. Vet. Clin. N. Am. Exot. Anim. Pract..

[4] Jekl V. (2021). Respiratory Disorders in Rabbits. Vet. Clin. N. Am. Exot. Anim. Pract..

[5] Varga M., Varga M. (2014). Chapter 11—Cardiorespiratory Disease. Textbook of Rabbit Medicine.

[6] Mancinelli E. (2019). Respiratory Disease in Rabbits. Practice.

[7] Grint N., Harcourt-Brown C. (2013). Anaesthesia. BSAVA Manual of Rabbit Surgery, Dentistry and Imaging.

[8] Lennox A.M., Mancinelli E. (2020). Respiratory Disease. Ferrets, Rabbits, and Rodents.

[9] Müllhaupt D., Wenger S., Kircher P., Pfammatter N., Hatt J.-M., Ohlerth S. (2017). Computed Tomography of the Thorax in Rabbits: A Prospective Study in Ten Clinically Healthy New Zealand White Rabbits. Acta Vet. Scand..

[10] Veraa S., Schoemaker N., Harcourt-Brown C. (2013). CT and MRI Scanning and Interpretation. BSAVA Manual of Rabbit Surgery, Dentistry and Imaging.

[11] Sargo R., Tomé I., Silva F., Ginja M. (2024). Evaluation of the Effects of Sedation and Anesthesia on Total Lung Volume and Attenuation in Rabbit Lung CT Exams. Animals.

[12] Capello V., Lennox A.M. (2011). Diagnostic Imaging of the Respiratory System in Exotic Companion Mammals. Vet. Clin. N. Am. Exot. Anim. Pract..

[13] Hunt T.D., Wallack S.T. (2021). Minimal Atelectasis and Poorly Aerated Lung on Thoracic CT Images of Normal Dogs Acquired under Sedation. Vet. Radiol. Ultrasound.

[14] Ali R.M.M., Ghonimy M.B.I. (2020). Semi-Quantitative CT Imaging in Improving Visualization of Faint Ground Glass Opacities Seen in Early/Mild Coronavirus (COVID-19) Cases. Egypt. J. Radiol. Nucl. Med..

[15] Kolta M.F.F., Abouheif M.A.A.-E.A.-E., Abd El-Mageed M.R. (2022). Semiquantitative CT Imaging as a Tool in Improving Detection of Ground Glass Patches in Patients with COVID-19 Pneumonia and for Better Follow-Up. Egypt. J. Radiol. Nucl. Med..

[16] Chen A., Karwoski R.A., Gierada D.S., Bartholmai B.J., Koo C.W. (2020). Quantitative CT Analysis of Diffuse Lung Disease. RadioGraphics.

[17] Sun D., Li X., Guo D., Wu L., Chen T., Fang Z., Chen L., Zeng W., Yang R. (2020). CT Quantitative Analysis and Its Relationship with Clinical Features for Assessing the Severity of Patients with COVID-19. Korean J. Radiol..

[18] Blazic I., Cogliati C., Flor N., Frija G., Kawooya M., Umbrello M., Ali S., Baranne M.-L., Cho Y.-J., Pitcher R. (2023). The Use of Lung Ultrasound in COVID-19. ERJ Open Res..

[19] Cole L., Pivetta M., Humm K. (2021). Diagnostic Accuracy of a Lung Ultrasound Protocol (Vet BLUE) for Detection of Pleural Fluid, Pneumothorax and Lung Pathology in Dogs and Cats. J. Small Anim. Pract..

[20] Ma H., Huang D., Guo L., Chen Q., Zhong W., Geng Q., Zhang M. (2016). Strong Correlation between Lung Ultrasound and Chest Computerized Tomography Imaging for the Detection of Acute Lung Injury/Acute Respiratory Distress Syndrome in Rats. J. Thorac. Dis..

[21] Rademacher N., Pariaut R., Pate J., Saelinger C., Kearney M.T., Gaschen L. (2014). Transthoracic Lung Ultrasound in Normal Dogs And Dogs with Cardiogenic Pulmonary Edema: A Pilot Study: Transthoracic Ultrasound of the Lungs in Dogs. Veter.-Radiol. Ultrasound.

[22] Armenise A. (2025). Point-of-Care Lung Ultrasound in Small Animal Emergency and Critical Care Medicine: A Clinical Review. Animals.

[23] Lin C.-H., Lo P.-Y., Lam M.-C., Wu H.-D. (2020). Usefulness of Chest Ultrasonography in Predicting Diagnosis in Non-Emergency Small Animal Patients with Lung Parenchymal and Pleural Disease. Front. Vet. Sci..

[24] Kraszewska K., Gajewski M., Boysen S., Buda N. (2025). Retrospective Evaluation of Subpleural Consolidations Using Lung Ultrasound in 634 Dogs and 347 Cats. Animals.

[25] Grune J., Beyhoff N., Hegemann N., Lauryn J.H., Kuebler W.M. (2020). From Bedside to Bench: Lung Ultrasound for the Assessment of Pulmonary Edema in Animal Models. Cell Tissue Res..

[26] Zhu Z., Lian X., Zeng Y., Wu W., Xu Z., Chen Y., Li J., Su X., Zeng L., Lv G. (2020). Point-of-Care Ultrasound—A New Option for Early Quantitative Assessment of Pulmonary Edema. Ultrasound Med. Biol..

[27] Lichtenstein D.A. (2009). Lung Ultrasound in the Critically Ill. J. Med. Ultrasound.

[28] Bujang M.A., Adnan T.H. (2016). Requirements for Minimum Sample Size for Sensitivity and Specificity Analysis. J. Clin. Diagn. Res..

[29] Lisciandro G.R., Fosgate G.T., Fulton R.M. (2014). Frequency and Number of Ultrasound Lung Rockets (b-Lines) Using a Regionally Based Lung Ultrasound Examination Named Vet Blue (Veterinary Bedside Lung Ultrasound Exam) in Dogs with Radiographically Normal Lung Findings. Vet. Radiol. Ultrasound.

[30] (2024). Slicer/SlicerLungCTAnalyzer. https://github.com/Slicer/SlicerLungCTAnalyzer/.

[31] Bumm R., Zaffino P., Lasso A., Estépar R.S.J., Pieper S., Wasserthal J., Spadea M.F., Latshang T., Kawel-Böhm N., Wäckerlin A. (2024). Artificial Intelligence (AI)-Assisted Chest Computer Tomography (CT) Insights: A Study on Intensive Care Unit (ICU) Admittance Trends in 78 Coronavirus Disease 2019 (COVID-19) Patients. J. Thorac. Dis..

[32] Reimegård E., Lee H.T.N., Westgren F. (2022). Prevalence of Lung Atelectasis in Sedated Dogs Examined with Computed Tomography. Acta Vet. Scand..

[33] Staffieri F., Franchini D., Carella G.L., Montanaro M.G., Valentini V., Driessen B., Grasso S., Crovace A. (2007). Computed Tomographic Analysis of the Effects of Two Inspired Oxygen Concentrations on Pulmonary Aeration in Anesthetized and Mechanically Ventilated Dogs. Am. J. Vet. Res..

[34] Vishwas K., Sandhyarani K., Madhuri D., Jeevanalatha M., Dhanalakshmi K. (2000). Incidence and Mortality Due to Respiratory Diseases in Rabbits. J. Entomol. Zool. Studies..

[35] Baker D.G. (1998). Natural Pathogens of Laboratory Mice, Rats, and Rabbits and Their Effects on Research. Clin. Microbiol. Rev..

[36] Hansell D.M., Bankier A.A., MacMahon H., McLoud T.C., Müller N.L., Remy J. (2008). Fleischner Society: Glossary of Terms for Thoracic Imaging. Radiology.

[37] Hewitt M.G., Miller W.T., Reilly T.J., Simpson S. (2014). The Relative Frequencies of Causes of Widespread Ground-Glass Opacity: A Retrospective Cohort. Eur. J. Radiol..

[38] Nemec S.F., Bankier A.A., Eisenberg R.L. (2013). Lower Lobe—Predominant Diseases of the Lung. Am. J. Roentgenol..

[39] Constantinescu R., Istrate A., Sumping J.C., Dye C., Schiborra F., Mortier J.R. (2023). Computed Tomographic Findings in Dogs with Suspected Aspiration Pneumonia: 38 Cases (2014–2019). J. Small Anim. Pract..

